# An updated view on horseradish peroxidases: recombinant production and biotechnological applications

**DOI:** 10.1007/s00253-014-6346-7

**Published:** 2015-01-11

**Authors:** Florian W. Krainer, Anton Glieder

**Affiliations:** Institute of Molecular Biotechnology, NAWI Graz, Graz University of Technology, Petersgasse 14, 8010 Graz, Austria

**Keywords:** Horseradish peroxidase, Plant peroxidase, Recombinant protein production, Diagnostics, Biosensor, Indole-3-acetic acid, Cancer treatment

## Abstract

Horseradish peroxidase has been the subject of scientific research for centuries. It has been used exhaustively as reporter enzyme in diagnostics and histochemistry and still plays a major role in these applications. Numerous studies have been conducted on the role of horseradish peroxidase in the plant and its catalytic mechanism. However, little progress has been made in its recombinant production. Until now, commercial preparations of horseradish peroxidase are still isolated from plant roots. These preparations are commonly mixtures of various isoenzymes of which only a small fraction has been described so far. The composition of isoenzymes in these mixed isolates is subjected to uncontrollable environmental conditions. Nowadays, horseradish peroxidase regains interest due to its broad applicability in the fields of medicine, life sciences, and biotechnology in cancer therapy, biosensor systems, bioremediation, and biocatalysis. These medically and commercially relevant applications, the recent discovery of new natural isoenzymes with different biochemical properties, as well as the challenges in recombinant production render this enzyme particularly interesting for future biotechnological solutions. Therefore, we reviewed previous studies as well as current developments with biotechnological emphasis on new applications and the major remaining biotechnological challenge—the efficient recombinant production of horseradish peroxidase enzymes.

## Introduction

**Fig. 1:**
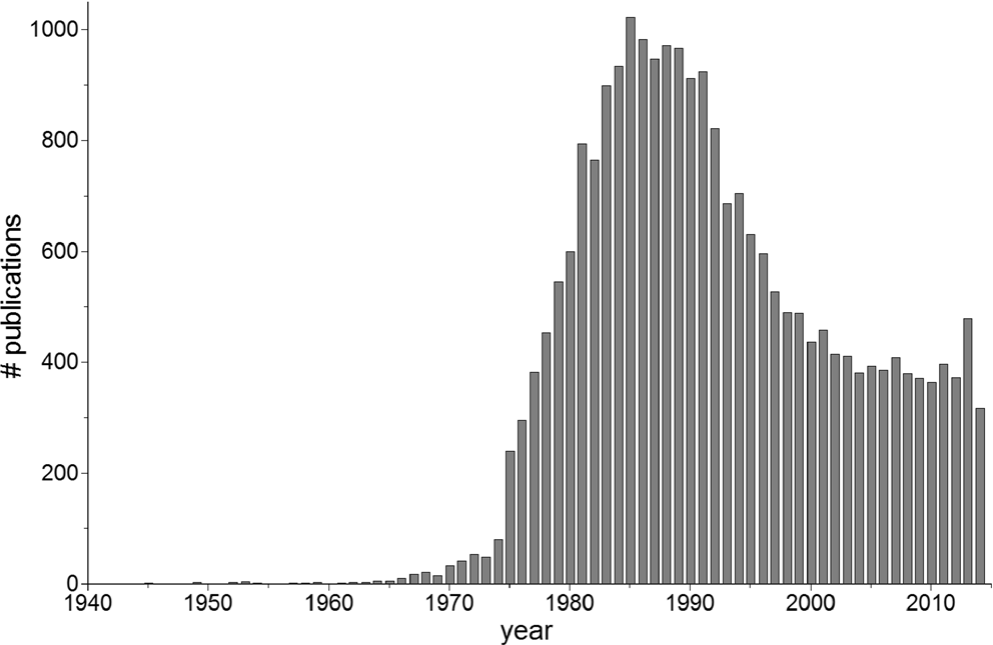
Scientific output on horseradish peroxidase over time. Publications involving horseradish peroxidase from 1940 to 2014 in the PubMed database (Aug 2014)

Peroxidases catalyze various oxidative reactions in which electrons are transferred to peroxide species (often H_2_O_2_) and substrate molecules are oxidized. These enzymes have been found in all living organisms, involved in a variety of biological processes. Peroxidase activity has been detected in a number of enzymes, predominantly classified to EC 1.11.1.7. Horseradish peroxidase (HRP) first appeared in scientific literature more than 200 years ago and has been the subject of numerous studies until now and ongoing. Scientific interest in HRP spiked in the late 1980s and early 1990s with the breakthrough of molecular diagnostics, molecular biology tool kits, and the publication of the first HRP gene (Fujiyama et al. 1988). Over the past 20 years, the number of publications involving HRP declined. Recently however, this decline not only flattened out but the number of publications rose by more than one third compared to the last 10 years, indicating new interest in this enzyme (Fig. 1).

HRP research typically deals with enzymological characterization, recombinant production in various expression systems, and to a large part with applications and their improvement by mutagenesis and chemical engineering. Until now, the vast majority of HRP research focused on one isoenzyme, C1A, neglecting the potential of other natural isoenzymes. Due to the discovery of new natural HRP isoenzymes (Näätsaari et al. 2014), an additional increase in the studies on HRP can be anticipated. In this review, we concisely highlight the crucial aspects of HRP research to give an overview of the state-of-the-art and particularly focus on the most recent strategies and developments of biotechnological relevance to provide access to the current challenges and opportunities.

## New horseradish peroxidase isoenzymes

The studies on peroxidase from horseradish date back as early as to the beginning of the nineteenth century. Already back in 1810, Planche reported the resin of Guaiacum plants turning blue upon contact with horseradish roots (Planche 1810). In the early 1950s, multiple components with peroxidase activity were found in horseradish, extending the number of peroxidase isoenzymes to 5 (Jermyn 1952; Jermyn and Thomas 1954). Moreover, seasonal variation in the relative amounts of these peroxidase components and differences in their reactivities towards different substrates were observed, underlining the hypothesis that the detected components were in fact separate isoenzymes rather than artifacts of a single peroxidase enzyme (Jermyn and Thomas 1954). In 1966, in the first of four papers on “peroxidase isoenzymes from horseradish roots” (Shannon et al. 1966; Kay et al. 1967; Strickland et al. 1968; Shih et al. 1971), the authors reported on the isolation, purification, and physical properties of seven distinct isoenzymes from horseradish. In the second publication of that same series, differences in pH optima, specific activities, and substrate affinities were described for these isoenzymes (Kay et al. 1967). In analogy to these previous findings, differences in kinetics and substrate profiles were found by comparing an acidic HRP isoenzyme with a basic one (Marklund et al. 1974). In 1977, a total of 42 HRP isoenzymes were detected by separating the peroxidase activities of three commercial preparations from horseradish by isoelectric focusing (Hoyle 1977). In the early 1980s, the isoenzymes E1–E6 and the five isoenzymes B1–B3, C1, and C2 were biochemically characterized (Aibara et al. 1981, 1982). Until recently however, only six nucleotide sequences of HRP isoenzymes were available from public databases. In 2014, we published a total of 28 sequences encoding enzymes with a secretory plant peroxidase domain that were found in a pyrosequenced transcriptome of *Armoracia rusticana*, horseradish, and showed diverging substrate profiles (Näätsaari et al. 2014). Natural HRP isoenzyme sequences that are currently available from public databases are shown in Table 1.

**Table 1:** Horseradish peroxidase isoenzymes

Isoenzyme	p*I*	MW kDa	GenBank	UniProt	References
C1A	5.7	38.8	M37156.1	P00433	(Fujiyama et al. 1988; Gajhede et al. 1997; Henriksen et al. 1998; Welinder 1976)
C1A	5.7	38.8	HE963800.1	K7ZWW6	(Näätsaari et al. 2014)
C1B	5.7	38.6	M37157.1	P15232	(Fujiyama et al. 1988)
C1B	5.7	38.6	HE963801.1	K7ZW26	(Näätsaari et al. 2014)
C1C	6.2	36.5	M60729.1	P15233	(Fujiyama et al. 1988)
25148.1 (C1C)	6.6	38.8	HE963802.1	K7ZWQ1	(Näätsaari et al. 2014)
25148.2 (C1D)	7.0	38.8	HE963803.1	K7ZW56	(Näätsaari et al. 2014)
C2	8.7	38.0	D90115.1	P17179	(Fujiyama et al. 1990)
04627 (C2)	8.7	38.0	HE963804.1	K7ZW02	(Näätsaari et al. 2014)
C3	7.5	38.2	D90116.1	P17180	(Fujiyama et al. 1990)
C3	7.5	38.2	HE963805.1	K7ZWW7	(Näätsaari et al. 2014)
A2	4.7	31.9	---	P80679	(Nielsen et al. 2001)
A2A	4.8	35.0	HE963806.1	K7ZW28	(Näätsaari et al. 2014)
A2B	4.8	35.1	HE963807.1	K7ZWQ2	(Näätsaari et al. 2014)
E5	9.1	33.7	–	P59121	(Morita et al. 1991)
E5	8.7	37.9	HE963808.1	K7ZW57	(Näätsaari et al. 2014)
N	7.5	35.1	X57564.1	Q42517	(Bartonek-Roxå et al. 1991)
01805	6.4	39.1	HE963809.1	K7ZW05	(Näätsaari et al. 2014)
22684.1	6.8	37.7	HE963810.1	K7ZWW8	(Näätsaari et al. 2014)
22684.2	6.3	37.8	HE963811.1	K7ZW29	(Näätsaari et al. 2014)
01350	8.7	34.3	HE963812.1	K7ZWQ3	(Näätsaari et al. 2014)
02021	9.6	35.8	HE963813.1	K7ZW58	(Näätsaari et al. 2014)
23190	8.4	39.4	HE963817.1	K7ZWQ4	(Näätsaari et al. 2014)
04663	4.4	37.2	HE963814.1	K7ZW09	(Näätsaari et al. 2014)
06351	6.9	34.6	HE963816.1	K7ZW31	(Näätsaari et al. 2014)
03523	8.9	35.6	HE963820.1	K7ZWX0	(Näätsaari et al. 2014)
05508	8.6	34.3	HE963815.1	K7ZWW9	(Näätsaari et al. 2014)
22489.1	8.8	34.8	HE963818.1	K7ZW59	(Näätsaari et al. 2014)
22489.2	8.8	34.8	HE963819.1	K7ZW11	(Näätsaari et al. 2014)
06117	5.7	36.4	HE963821.1	K7ZW32	(Näätsaari et al. 2014)
17517.1	9.6	35.1	HE963822.1	K7ZWQ5	(Näätsaari et al. 2014)
17517.2	9.6	35.1	HE963823.1	K7ZW60	(Näätsaari et al. 2014)
08562.1	9.0	36.1	HE963824.1	K7ZW15	(Näätsaari et al. 2014)
08562.4	9.0	36.1	HE963825.1	K7ZWX1	(Näätsaari et al. 2014)

Natural HRP isoenzymes are shown with their corresponding GenBank and UniProt database accession numbers and references. Isoelectric points and molecular weights were predicted with the Compute p*I*/Mw tool (Bjellqvist et al. 1993, 1994; Gasteiger et al. 2005) using unprocessed amino acid sequences (if available; the isoenzymes A2, P80689, and E5, P59121, were only available as processed peptides). For isoenzyme C1A, the amino acid at position 37 is Ile according to the GenBank entry M37156.1 but Tyr according to the GenBank entry HE963800.1 and the UniProt entries P00433 and K7ZWW6; calculations of pI and MW were performed with the latter sequence

When the gene structures of 73 class III peroxidases from the model plant *Arabidopsis thaliana* were studied, a conserved exon/intron structure of four exons and three introns was described (Tognolli et al. 2002). Similarly, we found this gene structure in 75 % of class III peroxidase sequences by comparing transcriptome and genome sequences of horseradish (Näätsaari et al. 2014).

The numerous peroxidase isoenzymes are thought to play specific physiological roles *in planta*. Peroxidase activity is detectable throughout the whole lifespan of a plant with a broad variety of reactions, which have been reviewed elsewhere (Passardi et al. 2005). However, only little information is available on the regulation of the expression of HRP isoenzymes. The recently identified HRP isoenzyme sequences (Näätsaari et al. 2014) will facilitate future studies on the expression patterns of individual isoenzymes in different plant tissues and in response to external stimuli. Also, due to their biochemical diversity, the large number of HRP isoenzymes constitutes a convenient toolbox of plant peroxidases from which an isoenzyme can be chosen that meets the requirements of an application best. For instance, the enzyme stability towards external factors (e.g. peroxide species, temperature) plays an essential role in biocatalysis and bioremediation (e.g. Kim et al. 2014a). An acidic HRP isoenzyme A2 has been found more stable towards H_2_O_2_ inactivation than an isoenzyme C (Hiner et al. 2001a). However, an isoenzyme E showed higher specific activity in oxalacetate oxidation than the isoenzymes A2 and C (Kay et al. 1967). Recombinant technology enables us to exploit and combine such features within the HRP toolbox, allowing for novel and improved biocatalysts in the near future.

## Structure and catalytic mechanism

Since enzymological features of HRP have been reviewed before exhaustively (e.g. Veitch and Smith 2001), we focus on the most recent studies and those features that are of relevance from a biotechnological perspective. So far, the majority of studies on HRPs have been performed on isoenzyme C1A; if not stated otherwise, all data mentioned here refer to this isoenzyme.

### Structural features of biotechnological relevance

**Fig. 2:**
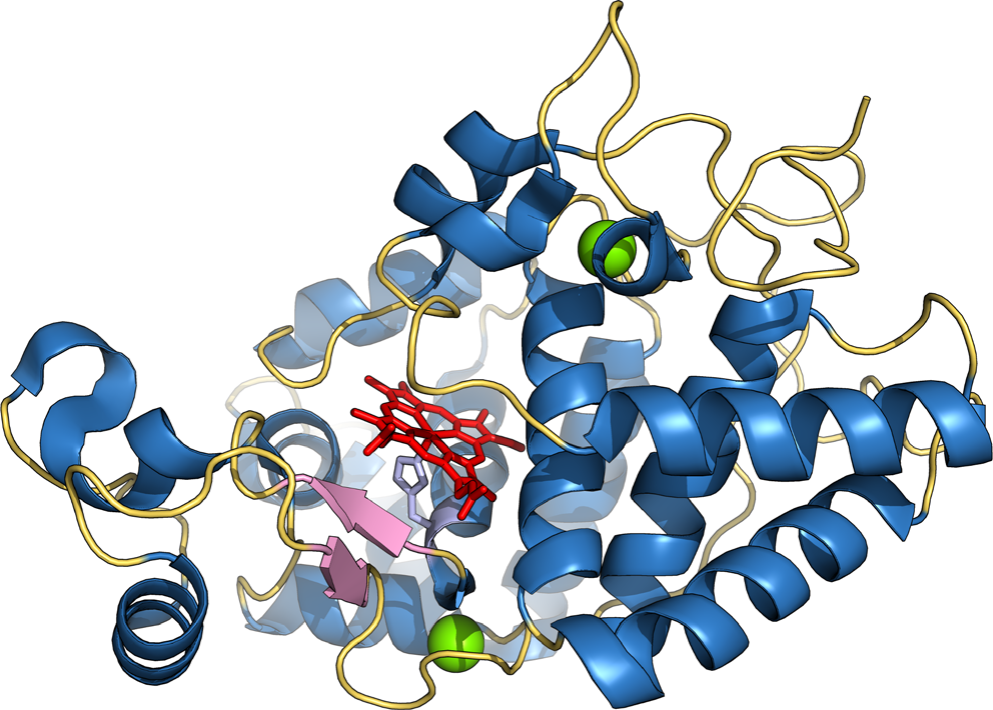
Structure of HRP C1A (PDB ID 1H5A). Helices and loops are shown in *blue* and *yellow*, respectively; one short β-sheet region is shown in *pink*. The two calcium ions are shown as *green spheres*. The heme group is shown in *red* and lies between the distal and the proximal domain; the proximal His170 residue (*light blue*) coordinates to the heme iron

HRP is a globular molecule with a predominantly α-helical secondary structure with the exception of one short β-sheet region (Fig. 2) (Gajhede et al. 1997). The HRP molecule is separated into a distal and a proximal region by an iron protoporphyrin IX cofactor, commonly referred to as the heme group. Heme is typically linked to HRP by a coordinate bond of the heme iron with a conserved His170 residue, facilitating the evaluation of the purity of a preparation by the ratio of A403 over A280, i.e. the absorbances of heme at the Soret band and of aromatic amino acids, respectively. This ratio is commonly referred to as the *Rz* value (originating from the German word *Reinheitszahl* which translates to “number of purity”) (Theorell et al. 1950). A *Rz* > 3.0 indicates a high relative heme content correlating with a high degree of enzyme purity. However, it has to be pointed out that different isoenzymes may yield different *Rz* values at comparable degrees of purity (Shannon et al. 1966). The availability of sufficient heme cofactor is a matter of consideration in recombinant production and can be tackled by media supplementation (e.g. de las Segura et al. 2005, Ryan and O’Fágáin (2008).

C1A contains nine Asn-X-Ser/Thr-X motives (X being any amino acid but Pro) as potential N glycosylation sites. *In planta*, all Asn residues of HRP C1A except for Asn286 are subjected to glycosylation (Welinder 1979). All glycosylated Asn residues are located on the surface of C1A (Gajhede et al. 1997). A total carbohydrate content of 21.8 % was reported for C1A; however, basic isoenzymes were found to have lower glycan contents (e.g. 0.8–4.2 % for the isoenzymes E3–E6) (Aibara et al. 1981; Yang et al. 1996). Chemical deglycosylation of C1A to a carbohydrate content of 5.5 % did not seem to affect the heme group but was described to cause structural changes and impaired enzymatic activity (Silva et al. 1990). In another study, all enzyme-linked carbohydrate structures were reduced by mild chemical deglycosylation to GlcNAc_2_, which remained attached to Asn. Both glycosylated and deglycosylated HRPs showed the same isoelectric point and absorbance spectrum and unaltered specific activity towards *o*-dianisidine, contrasting the previous findings. Only solubility in ammonium sulfate was found to be decreased upon deglycosylation (Tams and Welinder 1995). Further stability studies on deglycosylated HRP showed unaltered thermal stability, but threefold decreased kinetic stability in unfolding studies with guanidinium chloride (Tams and Welinder 1998). Additional studies will have to be conducted to conclusively determine the role of glycans on the biochemical properties of HRP. The recombinant production of individual isoenzymes from glycosylating expression hosts such as yeasts and non-glycosylating hosts such as *Escherichia coli* could be particularly useful for studies on HRP glycosylation.

Surface lysine residues are of particular interest for directed enzyme immobilization via covalent linkages. On the surface of HRP C1A, three (Lys174, Lys232, Lys241) out of six lysine residues were found accessible to chemical modifications (O’Brien et al. 2001).

In C1A, four disulfide bridges are formed between Cys41–121, Cys74–79, Cys127–331, and Cys207–239 (Welinder 1976), yet another feature that has to be kept in mind when it comes to choosing a host organism for recombinant production. Recombinant HRP from eukaryotic hosts such as *Pichia pastoris* does not require refolding to yield active enzyme, as opposed to HRP from *E. coli* which is typically produced as inclusion bodies (e.g. Smith et al. 1990).

### HRP catalysis and redox states

Peroxidative HRP catalysis can be roughly described by three consecutive reactions (Eqs. 1, 2, and 3) as established by Chance (1952) and George (1952, 1953).

1 $$ \begin{array}{lllllll}\mathrm{H}\mathrm{R}\mathrm{P}\hfill & +\hfill & {\mathrm{H}}_2{\mathrm{O}}_2\hfill & \to \hfill & \mathrm{H}\mathrm{R}\mathrm{P}\hbox{-} \mathrm{I}\hfill & +\hfill & {\mathrm{H}}_2\mathrm{O}\hfill \end{array} $$

2 $$ \begin{array}{lllllll}\mathrm{H}\mathrm{R}\mathrm{P}\hbox{-} \mathrm{I}\hfill & +\hfill & {\mathrm{AH}}_2\hfill & \to \hfill & \mathrm{H}\mathrm{R}\mathrm{P}\hbox{-} \mathrm{I}\mathrm{I}\hfill & +\hfill & {\mathrm{AH}}^{*}\hfill \end{array} $$

3 $$ \begin{array}{lllllllll}\mathrm{H}\mathrm{R}\mathrm{P}\hbox{-} \mathrm{I}\mathrm{I}\hfill & +\hfill & {\mathrm{AH}}_2\hfill & \to \hfill & \mathrm{H}\mathrm{R}\mathrm{P}\hfill & +\hfill & {\mathrm{AH}}^{*}\hfill & +\hfill & {\mathrm{H}}_2\mathrm{O}\hfill \end{array} $$

HRP, HRP-I, and HRP-II represent the enzyme in its resting state, a first intermediate state, compound I, and a second intermediate state, compound II, respectively. AH_2_ and AH^*^ are a reducing substrate and its oxidized radical species, respectively. The first step of the peroxidative cycle, compound I formation, was described by the Poulos-Kraut mechanism (Poulos and Kraut 1980). Recently, this step was suggested to happen as a “wet” mechanism via a single water molecule that allows formation of a ferric hydroperoxide intermediate, compound 0, based on QM/MM calculations (Derat and Shaik 2006; Derat et al. 2007; Vidossich et al. 2010). The O-O bond of compound 0 is then cleaved in accordance to the Poulos-Kraut mechanism, resulting in formation of compound I, which is reduced back to the enzyme’s resting state in two consecutive one-electron transfer reactions with two molecules of reducing substrate, as depicted above.

**Fig. 3:**
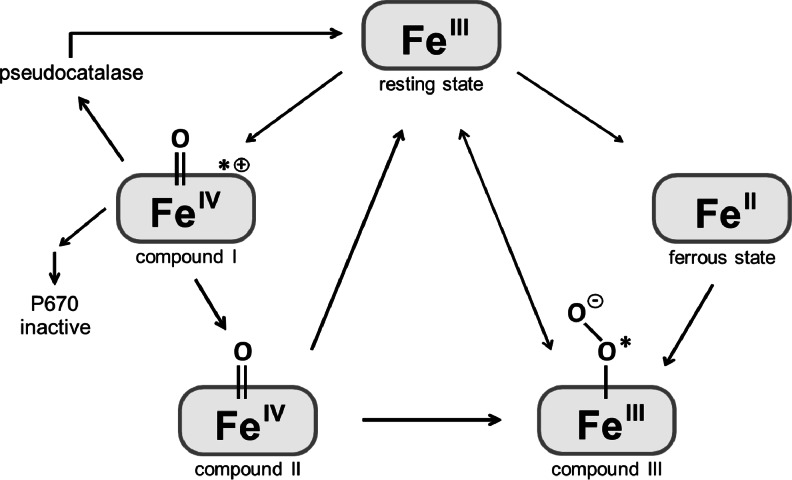
Schematic overview of HRP intermediate states. The peroxidative cycle starts with oxidation of the ferric resting state to an oxoferryl species plus a porphyrin-based π cation radical, compound I. Reduction of compound I by elimination of the π cation radical forms compound II which is reduced to return the enzyme to the resting state. Compound III (here shown as superoxide anion-binding ferric species) can be formed from a ferrous species, compound II, or directly from the resting state and slowly decays back to the latter. Upon peroxide excess, compound I can either react back to the resting state via a pseudocatalase activity, react further to compound II, or react to the inactive P670 species

Aside the three described oxidation states of HRP in the peroxidative cycle, two more oxidation states have been described: a ferrous species and compound III which can be described as either a dioxygen-binding ferrous species or an isoelectric ferric species binding a superoxide anion. The structures of these five intermediate states were solved in 2002 (Berglund et al. 2002) and are schematically depicted in Fig. 3.

In the absence of reducing substrates, excess peroxides react as reductants with compound I, giving rise to several spectroscopically distinct species (Keilin and Hartree 1951). In the case of H_2_O_2_, three pathways have to be considered subsequent to the formation of a compound I-H_2_O_2_ complex: (1) a catalase-like (i.e. pseudocatalase) two-electron reduction reaction that restores the enzyme to the resting state, (2) another catalytic pathway that leads to the formation of compound III which decays back to the resting state, (3) and a competing pathway that leads to the irreversible inactivation of the enzyme. The presence of a reducing substrate, as well as the two catalytic pathways, plays important roles in preventing enzyme inactivation by H_2_O_2_ (Arnao et al. 1990a, b). The enzymatic production of molecular oxygen via the pseudocatalase activity of HRP was described as a major protective mechanism against H_2_O_2_ inactivation (Hernández-Ruiz et al. 2001; Hiner et al. 2001b). However, this protective pathway is ineffective against peroxides other than H_2_O_2_. The inactivation pathway was described to first yield an intermediate with an absorbance band at 940 nm (P940) which further decays to an inactive verdohemoprotein species with an absorbance peak at 670 nm (P670) (Bagger et al. 1971; Vlasits et al. 2010). Studies comparing different isoenzymes showed basic isoenzymes to be more sensitive to inactivation than acidic isoenzymes under the tested conditions (Hiner et al. 1996, 2001a). Further studies on H_2_O_2_ susceptibility of natural HRP isoenzymes might yield valuable information for improving the oxidative stability of HRPs by rational design. A glycosylated isoenzyme C1A from plant was found to be twofold more resistant towards H_2_O_2_ inactivation compared to non-glycosylated recombinant C1A from *E. coli* and mutants thereof (Hiner et al. 1995), raising the issue on the role of HRP glycans once again. As a consequence, the recombinant HRP preparations from glycosylating hosts might be preferable over preparations from *E. coli* for applications requiring stable enzyme. However, the mutants Thr110Val, Lys232Asn, and Lys241Phe produced in another study in *E. coli* (Ryan and O’Fágáin 2007a) were found to be 25-, 18-, and 12-fold more stable towards H_2_O_2_ inactivation, respectively, than wild-type C1A. Suggested explanations for the findings were the removal of oxidizable groups or enhanced protective pseudocatalase activity. At this point, further studies are necessary to demonstrate a possible potentiation of the positive effects of these mutants from *E. coli* with the seemingly protective function of carbohydrate structures from a glycosylating production host.

## Recombinant production of horseradish peroxidases

Current commercial HRP preparations are typically isolated from horseradish roots as mixtures of several isoenzymes. The amount of individual isoenzymes in such a mixture depends on the *in planta* expression patterns of the respective isoenzymes (Jermyn and Thomas 1954). In general, it can be assumed that the quality and actual isoenzyme composition of any plant isolate depend on hard-to-predict and barely controllable environmental influences. To avoid this dependency on nature’s unpredictability and laborious steps to separate isoenzymes from one another, several organisms have been tested for their applicability as expression hosts for the recombinant production of HRP.

### Recombinant HRP production in *E. coli*

In 1988, a patent application was filed relating to the synthesis of a DNA sequence-encoding HRP C (Chiswell and Ortlepp 1988). The HRP gene was cloned into a vector and transformed to *E. coli* for expression. The staining of HRP expressing colonies with *N*,*N*,*N*′,*N*′-tetramethylphenylene diamine was performed to demonstrate the successful production of active HRP by *E. coli*. An anti-HRP antibody confirmed the identity of the produced peptide. In 1989, two further studies were published in which a recombinant HRP was successfully produced in *E. coli* (Burke et al. 1989; Ortlepp et al. 1989). In 1990, a procedure for intracellular expression of a synthetic C1A gene in *E. coli* and a protocol for refolding were described (Smith et al. 1990). The gene was based on a C1A sequence of 308 amino acids lacking the N-terminal signal peptide and the C-terminal propeptide (Welinder 1976; Fujiyama et al. 1988). To date, variations of this protocol for refolding of intracellularly produced HRP from inclusion bodies in *E. coli* are most commonly used for recombinant HRP production. Alternatively, some efforts have been taken to improve the recombinant production of active HRP in *E. coli* without the need for refolding. For instance, N-terminal fusion to a pelB leader peptide was performed for periplasmatic targeting to yield active enzyme (e.g. Grigorenko et al. 1999; Ryan and O’Fágáin 2008). However, the yield of holo-HRP was as low as 0.5 mg/L (Grigorenko et al. 1999). The coproduction of the DsbABCD proteins was suggested to support the correct formation of protein disulfide bonds when HRP was targeted to the periplasm of *E. coli* and to enhance the yield of active enzyme (Kurokawa et al. 2000). Nevertheless, it has to be pointed out that the production of secreted HRP in *E. coli* is still by far not suitable for biotechnological applications due to the extremely low yields. A general issue in recombinant production of active hemoproteins is heme availability. A common approach to improve heme supply is media supplementation with δ-aminolevulinic acid, a precursor molecule of the heme biosynthesis pathway (e.g. Ryan and O’Fágáin 2008). As an alternative, the coproduction of a heme receptor was reported to allow direct uptake of exogenously added heme by *E. coli* (Varnado and Goodwin 2004). Several more studies on improved HRP yields from *E. coli* have been published (e.g. Egorov et al. 1991; Grigorenko et al. 1999; Asad et al. 2013). However, the ultimately achievable yields from *E. coli* did not surpass 10 mg/L so far (Grigorenko et al. 1999), which cannot compete with the current isolation from plant and are thus not suitable for biotechnological applications. Therefore, most studies on recombinant HRP from *E. coli* deal with the production of C1A mutant variants to study the influence of specific amino acids on catalysis or stability. For instance, mutations of surface lysines resulted in augmented stabilities towards heat and solvent and higher enzymatic activity (Ryan and O’Fágáin 2008). In another study, arginine residues opposite the active site were mutated to lysines to form a batch of reactive groups suitable for increased and directed enzyme immobilization (Ryan and O’Fágáin 2007b). Notably, the stabilities of the studied mutants were found affected and a combination with stabilizing mutations will be required to link the favorable immobilization behavior with high enzyme stability. In order to stabilize recombinant HRP, selected residues were subjected to site-directed mutagenesis based on a sequence alignment of class III peroxidases (Ryan et al. 2008). However, the mutated consensus residues did not significantly improve thermal stability, indicating that this strategy could not be applied to class III peroxidases. In a recent follow-up study, an ancestral class III peroxidase was produced in *E. coli* and characterized (Loughran et al. 2014). This ancestral peroxidase was found twofold more resistant towards H_2_O_2_ inactivation than HRP C1A but less thermostable. In addition to the data on this ancestral peroxidase platform, comparative characterizations of the recently published extant HRP isoenzymes (Näätsaari et al. 2014) would provide additional data that could allow rational HRP design in the future after all. An overview of site-directed HRP mutants that have been characterized before 2006 was given in a previous review (Ryan et al. 2006). Thus, these studies will not be discussed here. It has to be pointed out that recombinantly produced HRP mutants from *E. coli* are commonly compared to recombinant wild-type enzyme from the same host but rarely to the plant-derived wild-type enzyme which might have yet again different characteristics, e.g. due its native glycosylation (Hiner et al. 1995). Hence, observed mutations on an unglycosylated mutant from *E. coli* might not be transferable to a glycosylated enzyme from a plant or yeast host (Hiner et al. 1995) and may require additional experimental data. Reevaluation of the advantageous mutations found in recombinant HRPs from *E. coli* in the context of a glycosylating production system might reveal a potentially additive benefit from enzyme-linked glycan structures.

### Recombinant HRP production in yeast systems

Mainly due to the presence of disulfide bridges, as well as to the considerable degree of glycosylation of native plant HRP, several studies have been performed on the production of recombinant HRP in eukaryotic expression systems. In 1992, *Saccharomyces cerevisiae* was used as a host for the secretory production of active hyperglycosylated HRP (Vlamis-Gardikas et al. 1992). Not only *S. cerevisiae* but also the methylotrophic yeast *P. pastoris* was used to study C1A mutants, generated by directed evolution to yield higher enzymatic activity and thermal stability (Morawski et al. 2000, 2001). An Asn175Ser mutation was found to increase thermal stability, presumably due to an additional hydrogen bond which was hypothesized to stabilize the enzyme’s heme cavity (Morawski et al. 2001). Conjugates of HRP with Fab fragments against atrazine were produced in *P. pastoris* and found to show functional antigen-binding properties (Koliasnikov et al. 2011). Hereby, the authors demonstrated the feasibility of recombinant HRP-antibody fusion proteins and provided an alternative to chemical conjugation procedures. Also, the use of HRP as a reporter enzyme in strain engineering and bioprocess studies with *P. pastoris* has been reported repeatedly (e.g. Hartner et al. 2008; Dietzsch et al. 2011a, b; Krainer et al. 2012, 2013a). Lately, we focused on the development and evaluation of purification protocols for different recombinant HRP isoenzyme preparations from *P. pastoris* (Spadiut et al. 2012; Krainer et al. 2013b). We performed two-step reverse chromatography purifications on 19 different isoenzymes and found considerable differences in both, purification efficiency and biochemical characteristics of the preparations. Strikingly, the number of N-glycosylation sites per isoenzyme appeared to affect both purification factor and recovery yield, indicating that yeast-type hypermannosylation of secreted HRP prevented interactions with the employed chromatography resins, thereby facilitating purification in reverse mode. A finding which is not only relevant for HRP purification but for secreted yeast-derived proteins in general (Krainer et al. 2013b).

In order to study the effect of N-glycosylation sites, recombinant C1A mutants were produced in *P. pastoris*, in which Asn residues of all N-glycosylation sites were systematically changed to either Asp, Gln, or Ser. The obtained mutant preparations were described to vary in their biochemical and physicochemical properties. Strikingly, a mutant preparation harboring mutations in all N-glycosylation sites showed a 300-fold reduction in catalytic activity compared to wild-type C1A (Capone et al. 2014). Regarding a medical application of recombinant HRP, the use of glycoengineered *P. pastoris* strains might be considered in order to allow human-type glycosylation.

By using a ΔAsn57-Ile70 deletion mutant of the prepro signal peptide of the *S. cerevisiae* mating factor α, which is commonly employed to facilitate enzyme secretion, HRP activity yields from *P. pastoris* could be increased by almost 60 % (Lin-Cereghino et al. 2013). Apparently, the passage through the secretory pathway describes a bottleneck in recombinant HRP production in *P. pastoris*, and additional engineering thereof is likely to further augment enzyme yields (e.g. Gasser et al. 2006; Guerfal et al. 2010).

Alternatively to *S. cerevisiae* and *P. pastoris*, a basidiomycete yeast strain, *Cryptococcus* sp. S-2, was recently used to produce more than 100 mg/L recombinant C1A in a fed-batch fermentation process. Expression was regulated by a xylose-inducible xylanase promoter, and a shortened xylanase signal peptide was used to mediate efficient secretion (Utashima et al. 2014). This strategy allowed for the highest yields of recombinant HRP so far. However, the employed yeast, *Cryptococcus* sp. S-2, is not “generally recognized as safe” (GRAS)—in contrast to *P. pastoris* (US Food and Drug Administration 2006). In the light of an application of HRP in medicine and for more convenient handling, the use of the latter might be reconsidered as soon as competitive yields can be achieved.

### Recombinant HRP production in insect systems

In 1992, an active secreted HRP was produced in cell cultures of insect SF9 cells (Hartmann and Ortiz de Montellano 1992). Thirteen years later, an improved protocol was published for HRP production in SF9 cells and subsequent purification. Up to 41.3 mg of active HRP was produced per liter of cell culture upon addition of hemin to the culture medium (de las Segura et al. 2005). By combining a poly-Arg and a poly-His tag fused to recombinant HRP from SF9 cells, the enzyme was purified 130-fold by cation-exchange chromatography at a yield of >98 % (Levin et al. 2005). Similar to *E. coli*, several mechanistic studies have been published on mutant HRPs produced in insect cell culture (Miller et al. 1995; Newmyer and Ortiz de Montellano 1995, 1996; Newmyer et al. 1996; Savenkova et al. 1996, 1998; Savenkova and Ortiz de Montellano 1998). Even though the yields from insect cells (40 mg/L; de las Segura et al. 2005) were fourfold higher than from *E. coli* (10 mg/L; Grigorenko et al. 1999), the yeast systems are still more promising (100 mg/L; Utashima et al. 2014) and allow more convenient handling than cell cultures. As an alternative to HRP production in insect cell cultures, an oral infective baculovirus was used for infection of lepidopteran larvae, and *Spodoptera frugiperda* larvae were found to produce the highest amount of HRP per larva (Romero et al. 2010, 2011). However, the competitiveness of this HRP production strategy in terms of handling and production efficiency remains yet to be demonstrated.

### Recombinant HRP production in plants

Plant hosts were described for the production of HRP as well, predominantly for studies on the physiological roles of peroxidases. In 1994, C1A was overexpressed in *Nicotiana tabacum*. The transformants were found to grow 20 % faster than wild-type plants (Kawaoka et al. 1994). Further studies on the impact of overexpressed HRP on growth and development of the tobacco plant emphasized the importance of subcellular peroxidase targeting (Heggie et al. 2005). For instance, tobacco plants showed increased axillary branching and decreased lignin deposition upon overexpression of C1A lacking a C-terminal vacuolar targeting peptide. However, these effects could not be observed when full length C1A was overexpressed. Additional studies (Matsui et al. 2003, 2006, 2011; Kis et al. 2004) were performed with transgenic tobacco to broaden the understanding on the regulation of vacuolar peroxidase targeting *in planta*. The expression of HRP not only in tobacco but also in hybrid aspen were found to increase growth rates in the transformed plants (Kawaoka et al. 2003). As a consequence, HRP overexpression in woody plants was suggested to increase biomass production for forestry and textile, pulp, and paper industries as a rather unconventional but nevertheless interesting application for HRP. Enhanced plant growth upon HRP overexpression might correlate with an altered metabolism of plant growth hormones such as indole-3-acetic acid (IAA) and remains to be demonstrated. In a recent publication (Walwyn et al. 2014), *Nicotiana benthamiana* was described for transient HRP expression, yielding 240 mg of HRP per kg of plant biomass. The authors calculated a yearly output of more than 5 kg of HRP by applying the described system at sufficiently large scale. Nevertheless, the handling of an appropriate microbial system might be more convenient and cost- and time-efficient, and further progress in microbial recombinant HRP production can be anticipated, considering the recent advances in recombinant HRP production in yeast systems (e.g. Lin-Cereghino et al. 2013; Utashima et al. 2014).

As an alternative to the production of HRP in a heterologous host, an in vitro production of HRP in horseradish hairy root cultures was reported (Uozumi et al. 1992; Flocco et al. 1998; Flocco and Giulietti 2003). However, this method takes a long time and yields a mixture of HRP isoenzymes that need to be separated from one another. Hence, it is not suitable for biotechnological application at this point.

### Remarks on current recombinant production systems

In conclusion, there are several hosts available that are suitable for the production of recombinant HRP (summarized in Table 2). Unfortunately, the comparability of the various hosts is complicated by the diversity of the reported yields (i.e. protein/volume, activity/volume, and activity/biomass). Hence, an absolute assessment of the production performance of a certain host is rather difficult from current literature. Moreover, there is variation in the quality of the produced HRP (purity, isoenzyme identity, glycosylation) and in the production costs of the respective hosts. These aspects will have to be taken into consideration in order to establish a recombinant production system. Recombinant HRP production at competitive yields is crucial in order to outmatch the current natural source. However, in light of the most recent promising results obtained with yeast host systems (e.g. Lin-Cereghino et al. 2013; Utashima et al. 2014), this goal appears achievable in the near future, allowing for well-defined enzyme preparations for the broad range of applications for the first time.

**Table 2:** Recombinant production systems for horseradish peroxidase

Expression host	Reported yields	Remarks	References
*Escherichia coli*	2–3 % of total protein	Refolded from inclusion bodies	(Smith et al. 1990)
	8–10 mg/L		(Grigorenko et al. 1999)
	0.5 mg/L	Targeted to periplasm; active	
*Saccharomyces cerevisiae* (baker’s yeast)	260 U/L/OD_600_	Secreted to supernatant; active	(Morawski et al. 2000)
*Pichia pastoris*	600 U/L/OD_600_		
	15 U/mL		(Hartner et al. 2008)
	1.66 U/g DCW/h		(Krainer et al. 2012)
*Cryptococcus* sp. S-2	110 mg/L	Optimized for codon usage and secretion	(Utashima et al. 2014)
*Spodoptera frugiperda* (fall armyworm)	41.3 mg/L	Produced in cell culture	(de las Segura et al. 2005)
	41 μg/larva	Produced in larvae	(Romero et al. 2010)
*Rachiplusia nu* (sunflower looper)	22 μg/larva		
*Nicotiana benthamiana*	240 mg/kg of plant biomass	Transient expression of isoenzyme C1A	(Walwyn et al. 2014)
*Nicotiana tabacum* (tobacco)	n/a	Growth studies	(Kawaoka et al. 1994)
*Populus* sp. (aspen)			(Kawaoka et al. 2003)
*Armoracia rusticana* (horseradish)		Produced in hairy root culture	(Flocco and Giulietti 2003)

Host organisms used for the expression of horseradish peroxidase are listed with their corresponding references. Yields from *Nicotiana tabacum*, *Populus* sp., and *Armoracia rusticana* were not available (*n*/*a*)

## Impact of recombinant technology on traditional and future HRP applications

Despite the aforementioned pitfalls in the isolation of HRP from horseradish roots and the challenges in recombinant production, numerous applications have been described for HRP in the fields of medicine, biotechnology, and life sciences. Several former examples thereof have been reviewed previously (Azevedo et al. 2003; Regalado et al. 2004). Here, we thus highlight only the most recent reports of applied HRP and emphasize the beneficial contributions of recombinant HRP to present-day applications.

### HRP as a reporter enzyme

Traditionally, HRP has been used exhaustively as a reporter enzyme, e.g. in histochemical stainings and diagnostic assays. For instance, HRP-conjugated secondary antibodies were used to detect HIV-1 envelope peptides expressed in cell culture via a cell-based ELISA (Veillette et al. 2014). HRP activity can either be detected by the formation of a chromogenic or fluorogenic product, or by an electrochemical signal due to the redox nature of HRP catalysis. The majority of studies on HRP in biosensor systems focuses on the detection of H_2_O_2_ (e.g. Kafi et al. 2008; Virel et al. 2010; Zhong et al. 2011; Ahammad et al. 2011). In recent years however, a considerable number of studies also dealt with the detection of other molecules, such as glucose (Alonso-Lomillo et al. 2005), ethanol (Azevedo et al. 2005), DNA and RNA (Fan et al. 2013; Tran et al. 2014; Saikrishnan et al. 2014), l-phenylalanine (Kubota et al. 2013), citrinin (Zachetti et al. 2013), pyrogallol and hydroquinone (Raghu et al. 2013), phenols (Kafi and Chen 2009; Liu et al. 2011), the milk allergen β-lactoglobulin (Ruiz-Valdepeñas Montiel et al. 2015), rotavirus titers (Li et al. 2014), and tumor markers (Chen et al. 2009; Kim et al. 2014b; Patris et al. 2014) via H_2_O_2_. Notably, studies on HRP applications of medical relevance are currently particularly prevalent. For example, HRP reporter activity was recently used for the quantification of protein kinase activity (Yin et al. 2015), which is of importance in cases of kinase-related drug discovery, therapy, and clinical diagnosis. In another study, HRP conjugates were used for the detection of DNA from the human pathogen *Mycobacterium tuberculosis* (Saikrishnan et al. 2014). A consistent enzyme quality is of particular relevance in medical diagnostics. These applications must not depend on fluctuating plant isolates and would therefore benefit greatly from a supply with consistent recombinant HRP.

To date, HRP is also a major component of high throughput assays in enzyme engineering, detecting H_2_O_2_ as a side product of biooxidants (Willies et al. 2012; Barber et al. 2014) or products of coupling reactions after biohydroxylations (Joo et al. 1999).

### HRP in biocatalysis

The applications of HRP in organic synthesis predominantly deal with polymerization reactions. For instance, HRP-catalyzed formation of poly(methyl methacrylate), a component of optical fibers, could be performed at ambient temperature (Kalra and Gross 2000). Also, polymerization reactions to form polystyrene (which is used e.g. as packaging material) from styrene and derivatives thereof were successfully performed with HRP (Singh et al. 2000). Moreover, HRP was used to form hydrogels, using phenolic derivatives of chitosan and polyvinyl alcohol (Sakai et al. 2014). By choosing the appropriate ratio of the two starting molecules, fibroblastic cell adhesion or growth of *E. coli* was either allowed or inhibited, respectively. Recently, the intrinsically conductive polymer poly(3,4-ethylenedioxythiophene) was synthesized in a two-step process involving HRP (Wang et al. 2014). However, enzyme inactivation by phenoxyl radicals describes a major limitation in oxidative polymerization reactions. A new study (Kim et al. 2014a) described a recombinantly produced quadruple mutant (Phe68Ala/Phe142Ala/Phe143Ala/Phe179Ala) to show improved stability towards radical inactivation at even faster substrate turnover than a wild-type enzyme, underlining yet again the so far largely untapped potential of recombinant HRPs in applications of industrial relevance.

Further HRP-catalyzed reactions of biocatalytical interest include oxidative dehydrogenation (Colonna et al. 1999), sulfoxidation (Colonna et al. 1992; Ozaki and Ortiz de Montellano 1994, 1995; Das et al. 2002; Yu and Klibanov 2006), and nitrogen oxidation (Kalliney and Zaks 1995; Boucher et al. 1996). In 2009, a yeast surface display strategy was adopted to screen point mutants of recombinant C1A. An Arg178Glu mutant showing enhanced enantioselectivity for phenol oxidation was identified (Antipov et al. 2009). Presumably, the combination of an efficient recombinant production system with enzyme engineering approaches will further improve and expand the use of HRP in biocatalysis.

### HRP in bioremediation systems

Beside the development of new electrochemical biosensor systems, most current publications on applied HRP deal with bioremediation systems to degrade synthetic dyes (Cheng et al. 2006; Ulson de Souza et al. 2007; da Silva et al. 2011; Bayramoglu et al. 2012; Malani et al. 2013; Preethi et al. 2013; Pereira et al. 2014) and to remove phenolic contaminants from wastewater (Bayramoğlu and Arica 2008; Bódalo et al. 2008; Alemzadeh and Nejati 2009; Vasileva et al. 2009; Li et al. 2013). Also in these applications, enzyme stability is a crucial factor. By modifying HRP with different polysaccharides, a starch-conjugated enzyme was recently found to show more than sixfold improved stability compared to unconjugated HRP. The enzymatic activity of starch-conjugated HRP was found unimpaired when applied for decolorization of bromophenol blue, demonstrating its applicability for wastewaters treatment (Kagliwal and Singhal 2014). The use of recombinant HRP mutants with improved stability behavior (e.g. Morawski et al. 2001; Ryan and O’Fágáin 2007a), as well as upcoming studies thereon are likely to yield further beneficial contributions in the near future. The broad substrate spectrum of HRP and the spontaneous polymerization of radicalized reaction products in particular facilitate the use of HRP in detoxification applications. Insoluble polymer molecules are easily separable from aqueous systems by filtration. Immobilization of HRP onto a suitable carrier surface facilitates reusability, it is known to affect enzyme activity and stability, and its optimization will certainly be the subject of future studies. In that regard, we recently filed a patent application on the use of immobilized recombinant HRP and variants thereof for the treatment of wastewater in order to remove e.g. endocrine-disrupting compounds such as synthetic estrogens (Kulterer et al. 2013). Also in terms of immobilization, the choice of an appropriate production host is crucial due to the absence or presence of glycan structures which could be exploited for convenient enzyme immobilization (Dalal and Gupta 2007) and affect enzyme stability (Hiner et al. 1995).

### Recent advances in cancer treatment using HRP in an enzyme-prodrug system

Since the late 1990s (Folkes et al. 1998, 1999), HRP has been studied in an enzyme/prodrug system for cancer treatment. The plant hormone IAA is part of the auxin metabolism and a natural substrate of HRP. It has long been known that oxidation of IAA by HRP does not require addition of H_2_O_2_ (Galston et al. 1953). However, due to the high number of partially unstable IAA derivatives and enzyme intermediates, the actual reaction mechanism is still not fully understood (Yamazaki and Yamazaki 1973; Kanofsky 1988; Gazarian et al. 1998). Targeting of HRP to cancer cells is crucial for the directed killing of these cells and can be achieved by either antibody-, polymer-, or gene-direct enzyme-prodrug therapy (ADEPT/PDEPT/GDEPT) (Folkes and Wardman 2001; Greco et al. 2001). The most recent explanation for the mechanism of HRP/IAA-induced cytotoxicity involves the induction of apoptosis due to the production of free radicals (Greco et al. 2002; de Melo et al. 2004; Kim et al. 2004). HRP/IAA-produced H_2_O_2_ was suggested to trigger the activation of the MAP kinases c-Jun N-terminal kinase and p38, caspase-3 activation, and poly(ADP-ribose) polymerase cleavage. The authors concluded that H_2_O_2_ is the key mediator of HRP/IAA-induced apoptosis (Kim et al. 2006a), involving the activation of a death receptor signaling pathway which is initiated by a H_2_O_2_-mediated increase in CD95 cell surface expression (Kim et al. 2006b). However, also other apoptotic pathways are likely to be involved in HRP/IAA-mediated cytotoxicity and additional studies will have to be conducted in order to unravel the overall molecular mechanism. In recent years, the HRP/IAA system has been tested successfully in vitro in various cancer cell lines of pancreatic cancer (Huang et al. 2005), lung carcinoma (Xu et al. 2011), urinary bladder carcinoma (Jeong et al. 2010), and hematopoietic tumor cells (Dalmazzo et al. 2011), as well as in vivo in mice (Xu et al. 2011; Dai et al. 2012). In a new study, chitosan nanoparticles encapsulating HRP were shown to induce cell death in a human breast cancer cell line. The encapsulation allowed for enhanced stability at 37 °C and in the presence of urea, compared to free enzyme (Cao et al. 2014). However, particularly with respect to this exciting medical application, a biotechnological approach would be considerably favorable over the current isolation procedure from plant roots to ensure a steady supply of recombinant HRP preparations at consistent quality via a controllable production process.

## Conclusions and outlook

HRP research has come a long way over the last two centuries. To date, HRP is a model enzyme for peroxidases. The numerous studies on its structural and mechanistic properties greatly broadened our understanding on peroxidase catalysis. On the other hand, plenty of questions remain unanswered so far, such as the identities and *in planta* roles of all different natural HRP isoenzymes, their biochemical properties, and their exploitation in the vast selection of potential applications. The use of an efficient recombinant expression system will greatly facilitate production processes of individual isoenzymes, which can then be evaluated for their suitability for any given application, an effort which is highly recommendable, regarding the diversity of the biochemical properties of the different isoenzymes. Also, further in-depth knowledge on the enzymology of different isoenzymes will facilitate rational design endeavors to generate mutant HRPs with tailor-made properties for any application, e.g. increased substrate affinity, solvent stability, peroxide resistance, or enantioselectivity. Regarding diagnostics, we now have the tools at hand to make use of modern recombinant technologies and a whole range of HRP isoenzymes with different characteristics to design tailored fusion proteins for a new and higher level of diagnostic kits with well-defined detection/reporter enzymes of consistent high quality. In light of a possible medical application of HRP in human, e.g. in ADEPT for cancer therapy, the glycan structures on the enzyme surface will have to be addressed not to infer with the human immune system. To avoid this issue, the use of engineered yeast strains that allow human-type glycosylation could be considered. On the other hand, a rapid clearance of those HRP molecules that did not bind to cancer cells might also be a desired effect.

Despite the astonishing number of studies on HRP, it seems that we have just scratched the surface. In light of the increasing demand for HRP due to the multitude of applications in life sciences and medicine, biotechnological state-of-the-art strategies will have to be adopted to allow a steady supply of well-defined HRP preparations at high quality.
